# PeGSTU58, a Glutathione S-Transferase from *Populus euphratica*, Enhances Salt and Drought Stress Tolerance in Transgenic *Arabidopsis*

**DOI:** 10.3390/ijms24119354

**Published:** 2023-05-27

**Authors:** Huijing Meng, Jinna Zhao, Yanfei Yang, Kehao Diao, Guangshun Zheng, Tao Li, Xinren Dai, Jianbo Li

**Affiliations:** 1 China National Permanent Scientific Research Base for Warm Temperate Zone Forestry of Jiulong Mountain, Experimental Centre of Forestry in North China, Chinese Academy of Forestry, Beijing 102300, China; 15938277261@163.com (H.M.); guangshunzheng@163.com (G.Z.); 2 College of Forestry, Shanxi Agricultural University, Taigu 030801, China; jinnazz@163.com (J.Z.); 13759225665@163.com (Y.Y.); m19903564319@163.com (K.D.); litao@sxau.edu.cn (T.L.); 3 State Key Laboratory of Tree Genetics and Breeding, Chinese Academy of Forestry, Beijing 100091, China; xinrend@caf.ac.cn

**Keywords:** *PeGSTU58*, *Populus*, *Arabidopsis*, salt stress, drought stress

## Abstract

Glutathione S-transferases (GSTs) play a crucial role in responding to abiotic stress and are an important target for research on plant stress tolerance mechanisms. *Populus euphratica* is a promising candidate species for investigating the abiotic tolerance mechanisms in woody plants. In our previous study, *PeGSTU58* was identified as being associated with seed salinity tolerance. In the present study, *PeGSTU58* was cloned from *P*. *euphratica* and functionally characterized. *PeGSTU58* encodes a Tau class GST and is located in both the cytoplasm and nucleus. Transgenic *Arabidopsis* overexpressing *PeGSTU58* displayed enhanced tolerance to salt and drought stress. Under salt and drought stress, the transgenic plants exhibited significantly higher activities of antioxidant enzymes, including SOD, POD, CAT, and GST, compared to the wild-type (WT) plants. Additionally, the expression levels of several stress-responsive genes, including *DREB2A*, *COR47*, *RD22*, *CYP8D11*, and *SOD1* were upregulated in *PeGSTU58* overexpression lines compared to those in WT *Arabidopsis* under salt and drought stress conditions. Furthermore, yeast one-hybrid assays and luciferase analysis showed that PebHLH35 can directly bind to the promoter region of *PeGSTU58* and activate its expression. These results indicated that *PeGSTU58* was involved in salt and drought stress tolerances by maintaining ROS homeostasis, and its expression was positively regulated by PebHLH35.

## 1. Introduction

Plants are frequently subjected to adverse environmental conditions, such as salt and drought, which limit their growth, development and productivity, ultimately resulting in significant economic losses. Such adverse environmental conditions induce the generation of reactive oxygen species (ROS). Excessive amounts of ROS can cause protein denaturation and mutations in nucleic acids, which can lead to oxidative damage and cell death [1,2]. To prevent damage, plants have evolved highly efficient antioxidant defense mechanisms that enable them to eliminate ROS and protect themselves against oxidative stress. Several ROS-scavenging enzymes have been identified, such as Glutathione S-transferases (GSTs), TRX, and GRX, and their functional role in stress response have has been elucidated [3,4].

The encoding of GSTs is accomplished by a vast gene family and the presence of these genes has been reported in various plant species. Studies have demonstrated that GSTs exhibit multifunctional roles, especially in cellular detoxification [5]. GSTs catalyze reduced glutathione (GSH) which is involved in plant defense system by scavenging free radicals and peroxides. GST proteins contain a GST N-domain which is involved in GSH binding and catalysis, and a GST C-domain involved in hydrophobic substrates binding [6,7]. Based on both protein sequence and structural similarity, plant GSTs can be segregated into eight distinct categories. Among the eight categories, Tau (GSTU) is a plant-specific class of the GST family and possesses a large number of members [8,9].

The role of GST in reducing abiotic stress has been verified in several *GSTU* genes across various species. In *Arabidopsis*, the expression of *AtGSTU19* is induced by abiotic stress. Overexpression of *AtGSTU19* conferred resistance to salt, drought, and methyl viologen stress tolerance [10]. Further studies found that *AtGSTU19 mutant* showed a higher amount of H_2_O_2_ in all root zones and confirmed that *AtGSTU19* plays a role in regulating root redox homeostasis and root meristem size [11]. The ectopic expression of rice *OsGSTU4* in *Arabidopsis* demonstrated better growth under salinity and oxidative stresses, achieved by reducing the accumulation of ROS and increasing GST activity in response to such stresses [12,13]. *GsGSTU42* and *GsGSTU24* from soybean conferred tolerance to flooding by improving the antioxidant capacity [14].

There have been some advances in understanding the regulatory mechanisms of *GSTUs* in response to abiotic stress. In transgenic plants overexpressing *OsGSTU4*, 63 genes showed significantly different expression levels compared to WT plants, with most of these genes being enriched in response to oxidative stress and defense response [13]. Overexpression of *AtGSTU19* was observed to enhance the expression of stress-related genes (*RD29A*, *KIN1*, and *COR15A*) [10]. The walnut *JrGSTTau1* gene has been shown to regulate stress-related genes, such as *NtGST*, *MnSOD*, *MAPK9*, and *CDPK15* [15]. GSTU8 has been found to interact with a calmodulin-like protein involved in regulating cold tolerance [16]. In addition, some transcription factors have been identified as regulators of *GST* expression. For example, CsWRKY48 was found to activate *CsGSTU8* expression by directly binding to the W-box region in its promoter in *Camellia sinensis*, and overexpression of *CsGSTU8* in *Arabidopsis* improved ROS scavenging capability and increased plant drought resistance [17].

The basic helix-loop-helix (bHLH) family is one of large superfamilies of transcription factors (TFs) in plants. Increasing numbers of bHLH family members have been found to play role in responding to abiotic stresses, such as drought, and salt stress [18,19]. The expression of *bHLH35*, for example, was notably elevated in response to salt stress in both *Lilium pumilum* [20]. The *AabHLH35* from *Anthurium andraeanum* increased the tolerance to drought and cold [21]. Ectopic expression of *TsPIP1;1* from *Thellungiella salsugineae* enhanced the salt tolerance in rice by upregulating the expression of *bHLH35* [22]. Overexpression of *Populus euphratica PebHLH35* increased tolerance to drought stress in transgenic *Arabidopsis* [23].

Although GSTs play a critical role in protecting herbaceous plants from oxidative stress under stress conditions, the characterization of their function and functional mechanism in perennial woody species remain scarce. *P. euphratica*, also known as the desert poplar, is mainly distributed in desert regions. Because of its extraordinary adaptation to salt and drought stress, *P*. *euphratica* is often used as a candidate species for investigating abiotic tolerance mechanisms in woody plants. In our previous genome-wide association study of *P. euphratica* seed salinity tolerance, the *PeGSTU58* gene was identified as one of the candidate genes [24]. This prompted us to explore its role in salt stress. Thus, *PeGSTU58* was isolated and functionally analyzed in the study. *PeGSTU58*, which is located in the cytoplasm and nucleus, encodes a Tau class GST. Overexpression of *PeGSTU58* increased tolerance to salinity and drought stress by improving ROS scavenging in transgenic *Arabidopsis*. Furthermore, the promoter of *PeGSTU58* can be directly bound by *PebHLH35*, which activates its expression. In summary, these results demonstrate that *PeGSTU58* plays a positive role in salt and drought stress resistance.

## 2. Results

### 2.1. Isolation and Characterization of PeGSTU58

**Figure 1:**
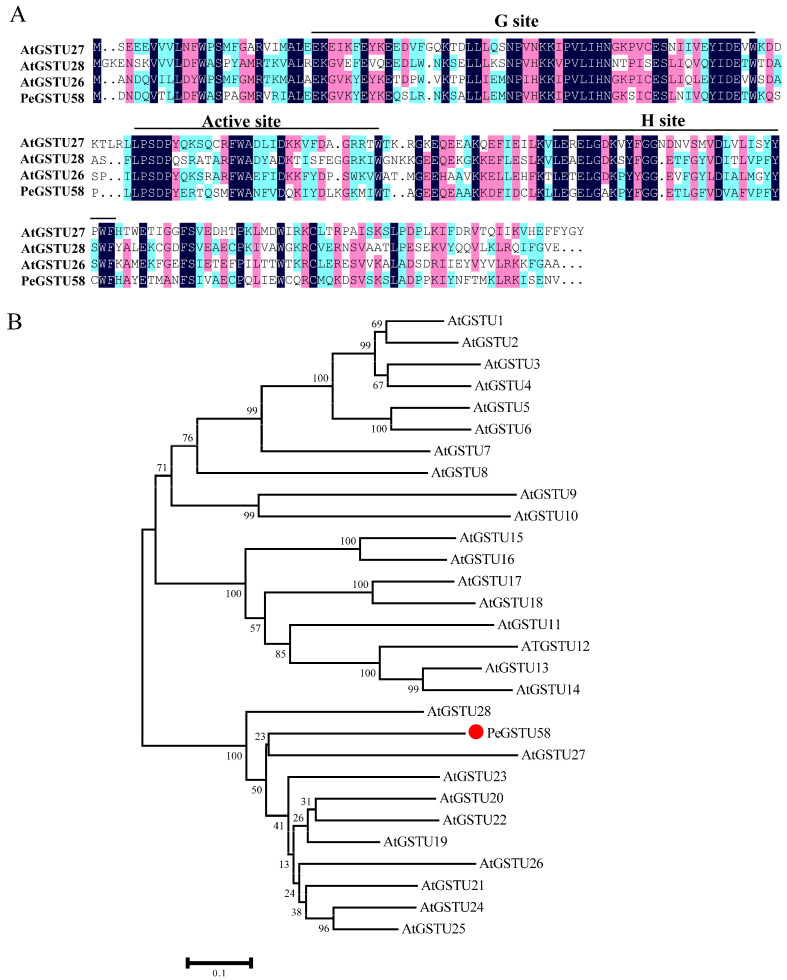
Phylogenetic analysis of PeGSTU58. (**A**) The phylogenetic relationship of PeGSTU58 with GSTUs from Arabidopsis. (**B**) Protein sequence multiple alignment of PeGSTU58 with its orthologous genes from Arabidopsis. The three consensus regions are underlined.

The coding sequence (CDS) of *PeGSTU58* was cloned based on the CCG006870 sequence information (Table S1). The CDS of *PeGSTU58* has a length of 660 bp and encodes a protein consisting of 219 amino acids with an expected weight of 25.3 kDa. The alignment of PeGSTU58 with its *Arabidopsis* orthologs was performed in order to ascertain its conserved domain and it revealed that PeGSTU58 contains a G site, an active site, and an H site (Figure 1A). In addition, MEGA 6.0 software [25] was utilized to perform a phylogenetic analysis of PeGSTU58 and Tau GST proteins from *Arabidopsis*, which revealed that PeGSTU58 exhibits a close relationship with AtGSTU27 (Figure 1B).

**Figure 2:**
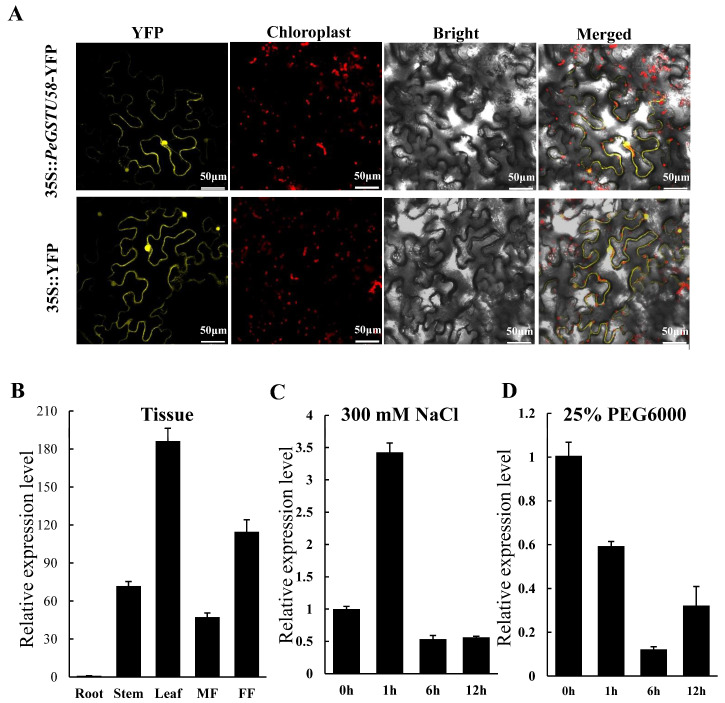
Subcellular localization and expression pattern of *PeGSTU58.* (**A**) The subcellular localization analysis of PeGSTU58 using tobacco leaves. (**B**) The expression analysis of *PeGSTU58* across various tissues. MF, male flower; FF, female flower. The expression of *PeGSTU58* in root was set to 1. (**C**,**D**) The expression analysis of *PeGSTU58* upon 300 mM NaCl (**C**) and 25% (*v*/*v*) PEG6000 treatment (**D**) in leaves. The expression of *PeGSTU58* at 0 h was set to 1.

To determine the subcellular localization of the PeGSTU58 protein, a *35S*::*PeGSTU58*-*YFP* construct was transiently expressed in tobacco leaf epidermal cells to detect its subcellular localization. After analysis, the fluorescence of the PeGSTU58 was detected in the cytoplasm and nucleus. This was similar to the fluorescent signal of the control YFP (Figure 2A).

To study the function of a gene, it is important to understand its expression pattern. In this study, the expression pattern of *PeGSTU58* in different tissues of *P. euphratica*, including male flower (MF), female flower (FF), leaf, stem, and root, and under stress conditions was investigated. The results showed that *PeGSTU58* was highly expressed in the leaf, followed by FF, while it exhibited low expression in the root (Figure 2B). Upon NaCl treatment, the expression of *PeGSTU58* exhibited an immediate increase, with a nearly three-fold increase observed after only one hour of exposure (Figure 2C). However, its expression significantly decreased at the 6 h and 12 h marks, reaching only 0.5 times that of the control. Under drought treatment, the expression level of *PeGSTU58* was observed to decline, reaching its lowest level, which was only 0.12-fold of the control at 6 h (Figure 2D). In addition, ARE and TC-rich repeats elements involved in the anaerobic induction, defense and stress responses were detected in the promoter of *PeGSTU58* (Figure S1). These results suggest that the expression of *PeGSTU58* is regulated by salt and drought stress.

### 2.2. Transgenic PeGSTU58 Arabidopsis Seedling Resistance to Salt and Drought Stress

**Figure 3:**
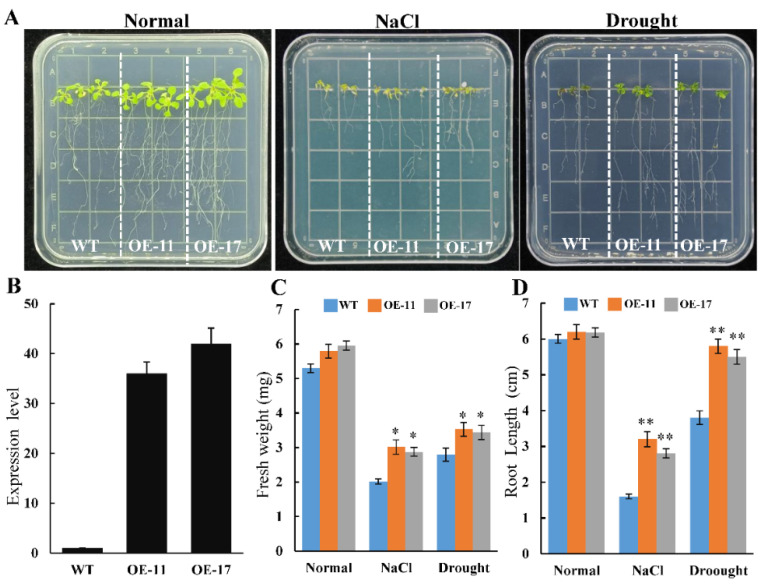
Drought and salt tolerance analysis of *PeGSTU58* transgenic seedlings in panel growth condition. (**A**) The image of *35S::PeGSTU58* (OE-11 and OE-17) and WT seedlings under normal condition, salt treatment, and drought treatment in plate. (**B**) qRT-PCR analysis of *PeGSTU58* expression level in *35S::PeGSTU58* (OE-11 and OE-17) and WT seedlings. (**C**,**D**) The fresh weight and root lengths of both WT and PeGSTU58-overexpressing seedlings were measured under normal conditions, salt, and drought stress treatments. Measurements of fresh weight and primary root lengths of *35S::PeGSTU58* (OE-11 and OE-17) and WT under normal, salt, and drought stress conditions. Significance test was conducted using Student’s *t*-test (* *p* < 0.05 and ** *p* < 0.01).

In our previous study, *PeGSTU58* was identified as related to seed salinity tolerance, suggesting its potential role in the salinity response [24]. To investigate the function of *PeGSTU58*, transgenic *Arabidopsis* lines with overexpressing *PeGSTU58* were generated. Two independent T3 homozygous *PeGSTU58*-transformed *Arabidopsis* lines (OE-11 and OE-17) with high transcript levels of *PeGSTU58* were used to evaluate the stress tolerance (Figure 3B).

Two developmental stages of seedling were chosen for stress tolerance analysis. Firstly, seedlings were transferred to 1/2 MS medium containing NaCl and mannitol to compare their growth conditions after one week of growth on 1/2 MS medium. Under normal condition, there were more roots, and branches in *PeGSTU58*-overexpression seedlings compared to WT. However, under NaCl and drought stress conditions, the growth of *PeGSTU58*-overexpression seedlings exhibited stronger resistance compared to WT. Specifically, under NaCl stress, the primary root length of WT seedlings was 1.6 cm, and the fresh weight was 2.33 mg, whereas *PeGSTU58*-overexpression seedlings had a primary root length of 2.8–3.2 cm and a fresh weight of approximately 3.03 mg. Similar results were observed under drought stress (Figure 3).

**Figure 4:**
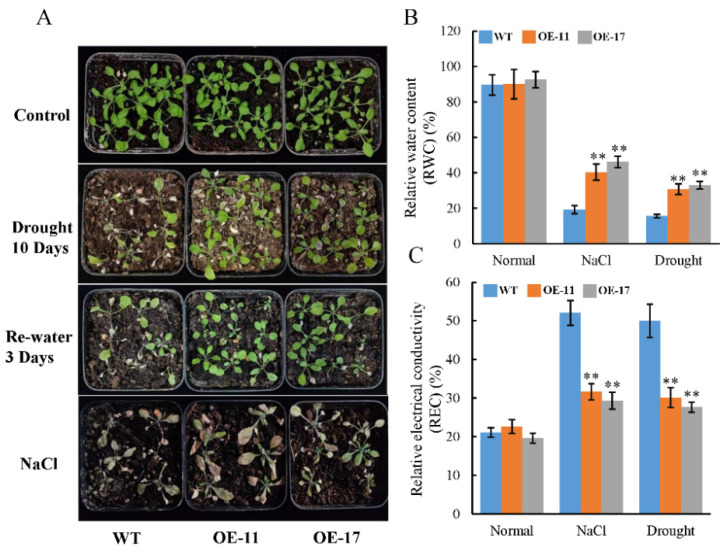
Transgenic *PeGSTU58 Arabidopsis* seedling resistance to salt and drought stress in soil. (**A**) *PeGSTU58*-overexpression and the WT seedlings were cultivated under normal growth conditions for two weeks in soil. Water was withheld for 10 days. This was followed by a rehydration period to examine the plants’ ability to withstand drought stress. For salt treatment, 30 mL of 200 mM NaCl was irrigated to the seedlings every 3 days for 3 weeks. Photographs were taken after each course of treatment. (**B**,**C**) The relative water content (RWC) and relative electrical conductivity (REC) of WT and the *PeGSTU58*-overexpression seedlings under control, salt, and drought stress was measured. The values presented herein result from an average calculation based on three separate replicates. Significance test was conducted using Student’s *t*-test (** *p* < 0.01).

Furthermore, two-week-old seedlings were initially grown in soil under normal conditions for two weeks, and were then subjected to either 200 mM NaCl or water withholding to assess their stress tolerance (Figure 4). After treatment, the growth of WT seedlings was severely inhibited. The *PeGSTU58* transgenic plants exhibited a significantly higher relative water content (RWC) of approximately 30% to 40% compared to WT plants which had an RWC of approximately 20% (Figure 4B). Relative electrical conductivity (REC) can be used to characterize the damage degree of plant cell membrane. Furthermore, the *PeGSTU58* transgenic plants showed an REC of around 30%, lower than that of the WT plants, which had an REC of approximately 50% under salt and drought stress (Figure 4C). These results indicate that overexpression of *PeGSTU58* alleviates growth inhibition of seedlings under salt and drought treatment.

### 2.3. PeGSTU58 Mediates ROS Scavenging Capability

**Figure 5:**
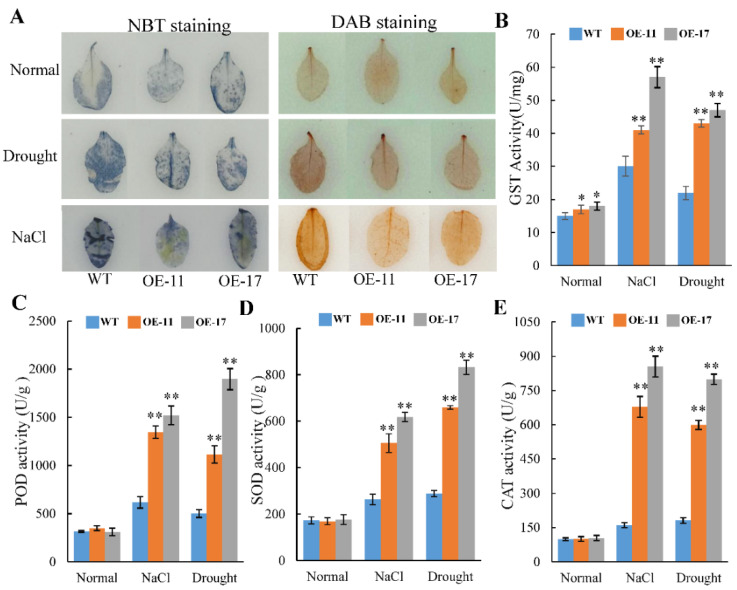
*PeGSTU58* mediates ROS scavenging capability. (**A**) NBT and DAB staining of leaves from *35S::PeGSTU58* (OE-11 and OE-17) and WT under normal and stress treatments. (**B**–**E**) The activities of GST (**B**), POD (**C**), SOD (**D**), and CAT (**E**) of WT and *PeGSTU58*-overexpression seedlings under normal and stress treatments. Significance test was conducted using Student’s *t*-test (* *p* < 0.05 and ** *p* < 0.01).

Abiotic stress can induce the production of ROS, including H_2_O_2_ and O^2−^ [26]. In order to assess the ROS content, 3, 3′-diaminobenzidine (DAB) and Nitroblue tetrazolium (NBT) stainings were used to measure the levels of H_2_O_2_ and O^2−^, respectively. Under normal growth conditions, there was no discernible difference in DAB or NBT staining between *PeGSTU58* transgenic and WT plants (Figure 5A). After salt and drought treatment, the staining signals in WT plants were darker than in the *PeGSTU58*-overexpression seedlings (Figure 5A), implying that there was a lower level of H_2_O_2_ and O^2−^ in the transgenic plants than in the WT plants under stress treatment.

Antioxidant enzymes play a crucial role in alleviating excess ROS. To investigate the mechanism of lower levels of H_2_O_2_ and O^2−^ in the transgenic plants, the activities of GST, superoxide dismutase (SOD), peroxidase (POD), and catalase (CAT) in *PeGSTU58*-overexpression seedlings and WT seedlings were measured under both normal and stress conditions. The results indicated that the transgenic plants displayed higher GST activity than WT plants. Moreover, the discrepancy in GST activity was found to have amplified after applying the salt and drought treatment (Figure 5B). Furthermore, while there was no significant disparity in the levels of POD, SOD, and CAT activities between *PeGSTU58*-overexpressing seedlings and WT seedlings under normal circumstances, under stressful conditions, the transgenic seedlings exhibited higher levels of POD, SOD, and CAT than that of the WT (Figure 5C,E). These findings suggest that overexpression of *PeGSTU58* can improve the tolerance to salt and drought by activating the antioxidant system.

### 2.4. The Expression of Stress-Related Genes Was Regulated in Transgenic PeGSTU58 Plants

**Figure 6:**
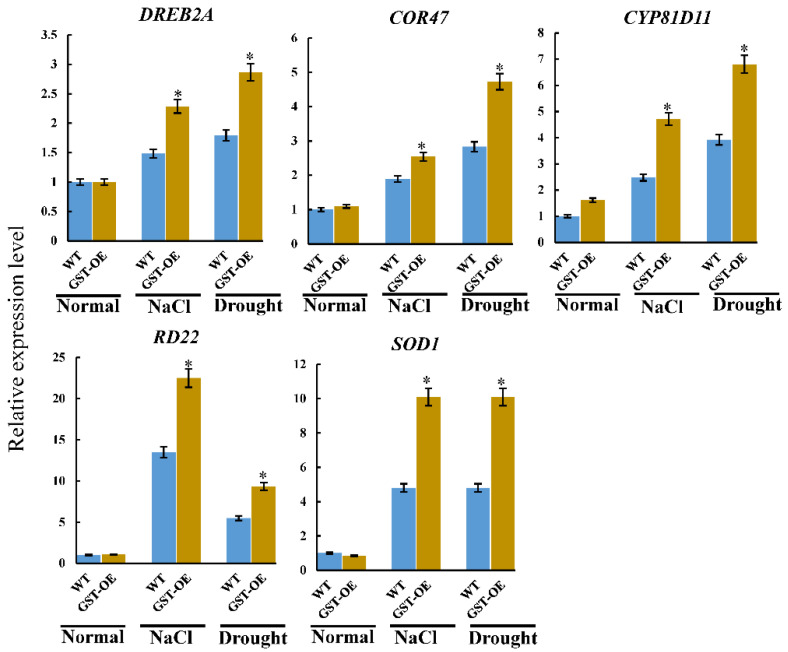
The expression levels of genes associated with stress in mixed two transgenic lines and WT plants. The expression level of each gene in the WT plant was set to 1. Significance test was conducted using Student’s *t*-test (* *p* < 0.05).

To clarify the functional mechanism of *PeGSTU58* in response to salt and drought stress, we examined the expression of stress-related genes previously reported to be upregulated in plants overexpressing *GSTU* [11]. These genes included *DREB2A*, *COR47*, *RD22*, *CYP8D11*, and *SOD1*, and their expression levels were analyzed in the leaves of both WT and the mixture of two transgenic lines under normal and stress conditions using qRT-PCR (Figure 6).

Under normal conditions, the expression level of *CYP8D11* was higher in *PeGSTU58*-overexpression seedlings than in WT, and the other genes showed no any significant difference. However, in the presence of salt and drought stress, these genes exhibited an upregulation in their expression levels (Figure 6). Notably, the expression levels were significantly higher in *PeGSTU58*-overexpression seedlings than in WT. Taken together, these results suggest that the expression of stress-related genes under salt and drought stress conditions was significantly enhanced in transgenic plants overexpressing *PeGSTU58*.

### 2.5. PebHLH35 Binds to the PeGSTU58 Promoter and Activates Its Expression

**Figure 7:**
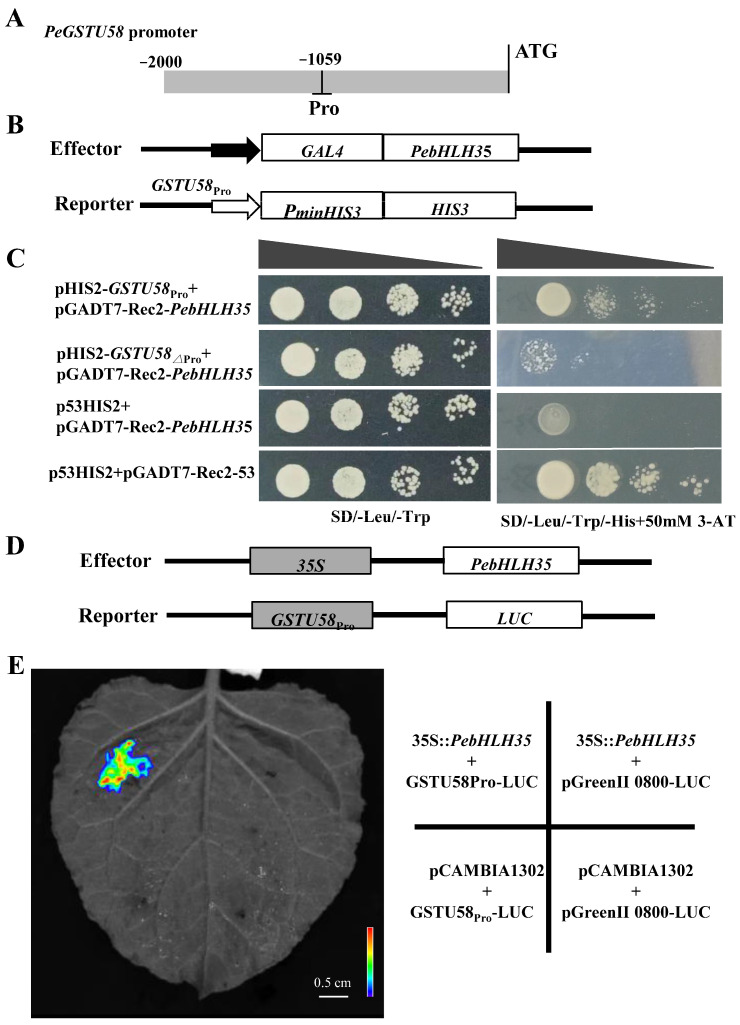
PebHLH35 bind to the *PeGSTU58* promoter and activates its expression. (**A**) The location of amplified promoter region (Pro) of PeGSTU58 in its promoter. (**B**) A diagram of vectors used for yeast one-hybrid analysis. (**C**) Yeast one-hybrid analysis of interactions between PebHLH35 and *PeGSTU58* promoter. The different combination of reporter and effector constructs were co-transformed into yeast Y187 cells, the positive control (p53HIS2 + pGAD-53), and the negative control (p53HIS2 + pGADT7-PebHLH35); *PeGSTU58*_∆*pro*_ means the promoter of *PeGSTU58* without “TCAACTTGA” motif. (**D**,**E**) A diagram of vectors used for transient dual-luciferase assays (**D**). The different combination of effector and reporter vectors were instantaneously co-transformed into *N. benthamiana* leaves. An image of *N. benthamiana* leaf with fluorescence was captured (**E**).

To further investigate the regulatory mechanism of *PeGSTU58*, PlantPAN v.3.0 [27] was used to predict the upstream regulators of *PeGSTU58*. The analysis suggested that putative bHLH proteins may combine with the promoter *PeGSTU58*. Among the bHLH proteins, PebHLH35 was confirmed to play roles in salt tolerance in a previous study [23] and it was predicated that PebHLH35 might bind to the “TCAACTTGA” motif in the promoter region of *PeGSTU58*. To confirm this, we generated a construct containing the promoter fragment of *PeGSTU58* with the “TCAACTTGA” motif (*pHis2-PeGSTU58_pro_*), without the “TCAACTTGA” motif (*pHis2-PeGSTU58*_∆*pro*_) and used them for yeast one-hybrid (Y1H) assay with *pGADT7-Rec2-PebHLH35*. The *p53-His2* and *pGADT7-p53* were used as positive controls and *p53-His2* and *pGADT7-Rec2-PebHLH35* were used as negative controls. All the yeast strains exhibited robust growth in a synthetic-defined (SD) medium (/-Trp/-Leu). The co-transformed yeast strains carrying *pHis2-PeGSTU58_pro_* and *pGADT7-Rec2-PebHLH35* were able to grow on the SD medium (/-Trp/-Leu/-His) containing 50 mm 3-AT (Figure 7C). In contrast, no growth was observed in the negative control group and the combination of *pHis2-PeGSTU58*_∆*pro*_ and *pGADT7-Rec2-PebHLH35* on the SD medium (/-Trp/-Leu/-His) containing 50 mm 3-AT (Figure 7C). These results indicate that PebHLH35 can bind to the promoter of *PeGSTU58* containing the “TCAACTTGA” motif.

To further verify the suppression or activation of *PeGSTU58* by PebHLH35, a luciferase (LUC) assay was carried out for the transient expression in tobacco leaves. The leaves were co-infiltrated with *35S::PebHLH35* and *PeGSTU58pro::LUC* constructs (Figure 7D). Strong fluorescent signals were observed in the tobacco leaves that were co-infiltrated with both constructs (Figure 7E), while no fluorescence was observed in the control samples. These results demonstrate that PebHLH35 is capable of directly binding to the *PeGSTU58* promoter and activating its expression.

## 3. Discussion

GSTs are a diverse and large family of enzymes that play a pivotal role in plant stress tolerance by protecting against oxidative damage [26]. The functions of GSTs have been extensively studied in various plant species, including *Arabidopsis* [28], rice [29], *Camellia sinensis* [17], barley [30], potato [31], pepper [32], *Vitis vinifera* [33], *Tamarix hispida* [34], maize [35], and *Citrus sinensis* [36]. In *P. euphratica*, only *PeGSTF4* has been investigated for its positive role in stress tolerance [37]. Based on our previous study [24], *PeGSTU58* was identified as a candidate gene involved in salinity tolerance in *P. euphratica*, and its regulatory function in response to abiotic stress was investigated in this study.

Phylogenetic analysis indicated that PeGSTU58 is closely related to AtGSTU27 and belongs to the Tau class (Figure 1), suggesting that PeGSTU58 may have conserved roles with the members of the Tau class and may be involved in abiotic stress responses. Protein structure analysis showed that PeGSTU58 has a conserved GSH binding site (Figure 1A), which plays a crucial role in the conjugation of the GSH moiety, implying that PeGSTU58 may have the ability to catalyze GSH. This speculation was confirmed by the higher GST activity observed in *PeGSTU58*-overexpression plants compared to WT plants under normal and drought stress conditions (Figure 5A).

Gaining insight into the subcellular localization of proteins is advantageous for the functional analysis of genes. MdGSTU12 from *Malus domestica* was found to be located in the cytoplasm where it functions as a cytoplasmic transporter and contributes to the accumulation of anthocyanin [38]. OsGSTU4 was observed in both the cytoplasm and nucleus where it enhances the expression of genes involved in stress responses and cellular detoxification processes [12]. The dual localization of PeGSTU58 in the cytoplasm and nucleus (Figure 2A) suggests that it may have multiple roles in cellular processes. In addition to its potential function as a transporter within the cytoplasm, the nuclear localization of PeGSTU58 implies that it may play a role in regulating transcription. Additionally, the ability of *PeGSTU58* to regulate the expression of *DREB2A* and *RD22* genes in *PeGSTU58*-overexpression plants (Figure 6) suggests that it may function as a transcriptional regulator. However, the specific molecular mechanisms through which PeGSTU58 controls gene expression require further investigation. Moreover, further studies are necessary to identify the interacting partners and target genes of PeGSTU58 in both cytoplasmic and nuclear compartments to fully understand its biological functions.

*PeGSTU58*-overexpression transgenic plants were employed to evaluate the function of PeGSTU58. The transgenic plants showed superior growth under salt and drought conditions, as evidenced by their increased root length and fresh weight, compared to WT plants. Plant stress leads to ROS accumulation. Excess ROS is then eliminated by the enzymatic defense systems. In this study, the activities of POD, SOD, and CAT increased and the accumulation of ROS was reduced when *PeGSTU58*-overexpressed plants were exposed to salt and drought stress, suggesting a positive role of PeGSTU58 in ROS scavenging. These results are consistent with previous research on the functional properties of GSTUs [12,13,14] and indicate that GSTUs have a crucial role in plant stress responses by enhancing the activities of antioxidant enzymes.

Under abiotic stress, GST expression can be consistently altered, with some GSTs exhibiting reduced expression. For example, in *T. hispida*, the expression of *ThGSTZ1* was significantly reduced under salt and drought stress, and its overexpression enhanced the plant’s ability to withstand these stresses [34]. In another study, the expression of *GsGSTU13* was induced after exposure to NaCl for 1 h, but its expression was later inhibited from 3 to 12 h [39]. However, many *GST* genes activated by abiotic stress have been investigated and found to play a positive role in stress response [11,12,17]. In our study, we found that the expression of *PeGSTU58* was induced in the early stage of salt treatment, but inhibited in the later stage under drought. The findings imply that the *GST* superfamily’s transcript regulation is influenced by numerous different mechanisms.

In the process of signal transduction in response to abiotic stress, various transcription factors (TFs), such as ERF [29] and WRKY [17], bind to the promoters of GSTs. This binding ultimately leads to the modulation of GST expression, which enables an organism to effectively respond to the external environment. In addition, one GST also can be regulated by multiple TFs. For example, in *Juglans regia*, *JrGSTTau1* can be activated by JrMYC2, JrDof1, and JrWRKY7 in response to osmotic stress [15]. In response to oxidative stress and extreme temperature, AtERF2 positively modulates the expression of *AtGST11*, whereas homeobox protein 6 exerts a negative regulatory effect [40]. In our study, we detected several *cis*-elements in the promoter of *PeGSTU58* (Figure S1) and predicted TFs that may regulate its expression. PebHLH35 was confirmed to be directly bind to the promoter of *PeGSTU58* using yeast one-hybrid assays and luciferase analysis. Previous study has shown that increased expression of *PebHLH35* leads to improved resistance against water-deficit stress [23]. Therefore, we propose that *PeGSTU58* acts as a positive factor in abiotic stress tolerance, at least in part, being regulated by PebHLH35.

In summary, *PeGSTU58* was isolated and characterized from *P. euphratica* as a positive regulator of salt and drought stress. Overexpression of *PeGSTU58* resulted in significant enhancement of salt and drought tolerance in plants by reducing the levels of ROS and elevating the activity of antioxidant enzymes. Furthermore, *PeGSTU58* overexpression was found to induce the expression of genes associated with stress response. In addition, PebHLH35 was identified as a potential upstream regulator of *PeGSTU58*. The investigation provides a foundation for gaining deeper insights into the function of *PeGSTU58* and the underlying molecular mechanism of salt and drought tolerance in *P. euphratica.*

## 4. Materials and Methods

### 4.1. Plant Material and Treatments

*A. thaliana* (ecotype: Col-0) was selected as wild-type (WT) and used as a transgene receptor. The *Arabidopsis* seeds underwent a sterilization procedure that involved two rounds of exposure to 75% ethanol and a subsequent single exposure to 100% ethanol. After the sterilized seeds were dry, they were sown in a plate containing 1/2 MS medium and subsequently stored at 4 °C for 3 days. The plate were cultivated under 20–22 °C, while being exposed to a light and dark cycle of 16 h and 8 h, respectively. Two weeks after germination, the seedlings were transplanted into soil for further cultivation.

### 4.2. A. thaliana Transformation and Isolation of Transformed Plants

The CDS of *PeGSTU58* was cloned and constructed into over-expression vector PMDC32, which was driven by a 35S promoter, labelled as *35S::PeGSTU58*. The floral dip method was employed for *Agrobacterium*-mediated *A. thaliana* transformation [41]. Upon screening the T0 seeds in a selection medium containing 25 mg/L hygromycin, over 30 transgenic lines were successfully procured. After qRT-PCR analysis, two independent transgenic lines with high abundance of *PeGSTU58* were used for further study.

### 4.3. RNA Extraction and qRT-PCR Assays

The RNA was isolated using the RNeasy Plant kit (Tiangen, Beijing, China) and qRT-PCR were carried out in accordance with their protocol. *AtActin7* was chosen as a reference gene for gene expression analysis in *A. thaliana. PeActin, PeEF1α,* and *PeHIS* were selected as reference genes for different tissue types, NaCl treatment, and drought treatment in *P. euphratica* [42]. For the qRT-PCR analysis, each sample was subjected to three biological replicates and four technical replicates. The primers were listed in Table S2.

### 4.4. Subcellular Localization of PeGSTU58

To create the 35S::*PeGSTU58*-YFP construct, the lack of a stop codon in the CDS of *PeGSTU58* was incorporated into the pEarleyGate101 vector. This construct was then transformed into *Agrobacteriu*. After infiltration of *Agrobacterium* to tobacco leaves for 2 days, the fluorescence signal was observed using a LSM 510 confocal laser scanning microscope.

### 4.5. Stress Tolerance Analysis

For the salt and drought tolerance experiments, one-week-old seedlings of *35S::PeGSTU58* and WT were cultivated on 1/2 MS medium containing 150 mM NaCl or 200 mM mannitol. The fresh weight and primary root length were assessed after a treatment period of one week.

In addition, two-week-old seedlings of *35S::PeGSTU58* and WT grown on normal medium for two weeks, were planted in soil. After they were grown in soil for four weeks under normal conditions, the seedlings were subjected to salt treatment by administering 30 mL of 200 mM NaCl through irrigation. After two weeks of salt treatment, the seedlings were irrigated with pure water for seven days. For drought treatment, the seedlings were grown in well-watered soil for two weeks. Irrigation was then withheld until the seedlings began to wilt. After this, they were well watered for a further week.

### 4.6. Physiological Measurements and Histochemical Assays

Fresh leaves of *35S::PeGSTU58* and WT under normal, drought, and salt stress condition were collected for physiological measurement including REC, RWC, and enzyme activities. The REC and RWC were measured according to a previous method [43]. The activities of antioxidant enzymes (SOD, POD, CAT, and GST) were measured using the test kit (Solarbio, Beijing, China) in accordance with protocols. Three replicates of each index measurement were taken.

For DAB staining, the DAB staining solution with 1 mg/mL concentration was adjusted to pH 3.8 with dilute hydrochloric acid. The obtained leaves were placed in the DAB staining solution and evacuated in a vacuum pump for 20 min. They were then placed in a dark place at 25 °C for 6–8 h [44]. For NBT staining, the NBT staining solution with 0.5 mg/mL concentration was adjusted to pH 7.8. The obtained leaves were placed in the NBT staining solution and vacuumed for 20 min, then placed in a dark place at 25 °C for 1 h [45]. After staining, the leaves were decolorized in a 95 °C water bath in 95% anhydrous ethanol. Lastly, images were captured using a Zeiss Axio Imager A1 microscope (Carl Zeiss, Jena, Germany).

### 4.7. Prediction the Upstream Regulators of PeGSTU58

The promoter sequence of *PeGSTU58* was uploaded to the PlantPAN v.3.0 (http://plantpan.itps.ncku.edu.tw/promoter.php accessed on 12 December 2021) online software to predict the upstream regulators of PeGSTU58, according to the previous description [27].

### 4.8. Yeast One-Hybrid (Y1H) Assay

The promoter fragment of *PeGSTU58* and the full-length CDS of PebHLH35 were inserted into pGADT7-Rec2 and pHIS2 vector to generate the recombinant plasmid pHis2-PeGSTU58_pro_ and pGADT7-Rec2-PebHLH35, respectively. The plasmid pHis2-PeGSTU58_pro_ and pGADT7-Rec2-PebHLH35 were co-transformed into Y187 yeast strain and screened on the SD/-Leu/-Trp deficient medium. The successfully transformed yeast strains were proliferated and cultured to an OD value of 0.8, and then diluted 10, 100, and 1000 times and dripped onto the SD/-Leu/-Trp/-His containing 50 mM 3-AT, respectively.

### 4.9. Transient Dual-Luciferase Assays

The luciferase reporter construct (*PeGSTU58*pro::Luc) was generated by inserting the promoter fragment of *PeGSTU58* into the pGreenII 0800-LUC vector. The effector vector (*35S*::*PebHLH35*) was produced by incorporating *PebHLH35* into the pCAMBIA 1302 vector. The vectors were then transformed into *Agrobacterium* and were subsequently co-expressed in tobacco leaves in a transient manner. After being cultured in an incubator under control conditions for 48 h, the relative LUC activity levels fluorescent were measured and images were captured using plant view 100 (BLT, Xi’an, China).

### 4.10. Statistical Analyses

Statistical analyses were conducted using SPSS statistics 19.0 (SPSS Inc., Chicago, IL, USA). The data underwent a comparison process using *t* test. In the event that the *p* value was below 0.05, the variances were regarded as significant.

## Data Availability

Data generated in the current work is provided in the manuscript.

## Supplementary Materials

The following supporting information can be downloaded at: https://www.mdpi.com/article/10.3390/ijms24119354/s1.
