# Supplementary figures and images for: PeGSTU58, a Glutathione S-Transferase from Populus euphratica, Enhances Salt and Drought Stress Tolerance in Transgenic Arabidopsis
Source: Int J Mol Sci. 2023 May 27;24(11):9354. doi: 10.3390/ijms24119354 (PMC10253091; doi:10.3390/ijms24119354)

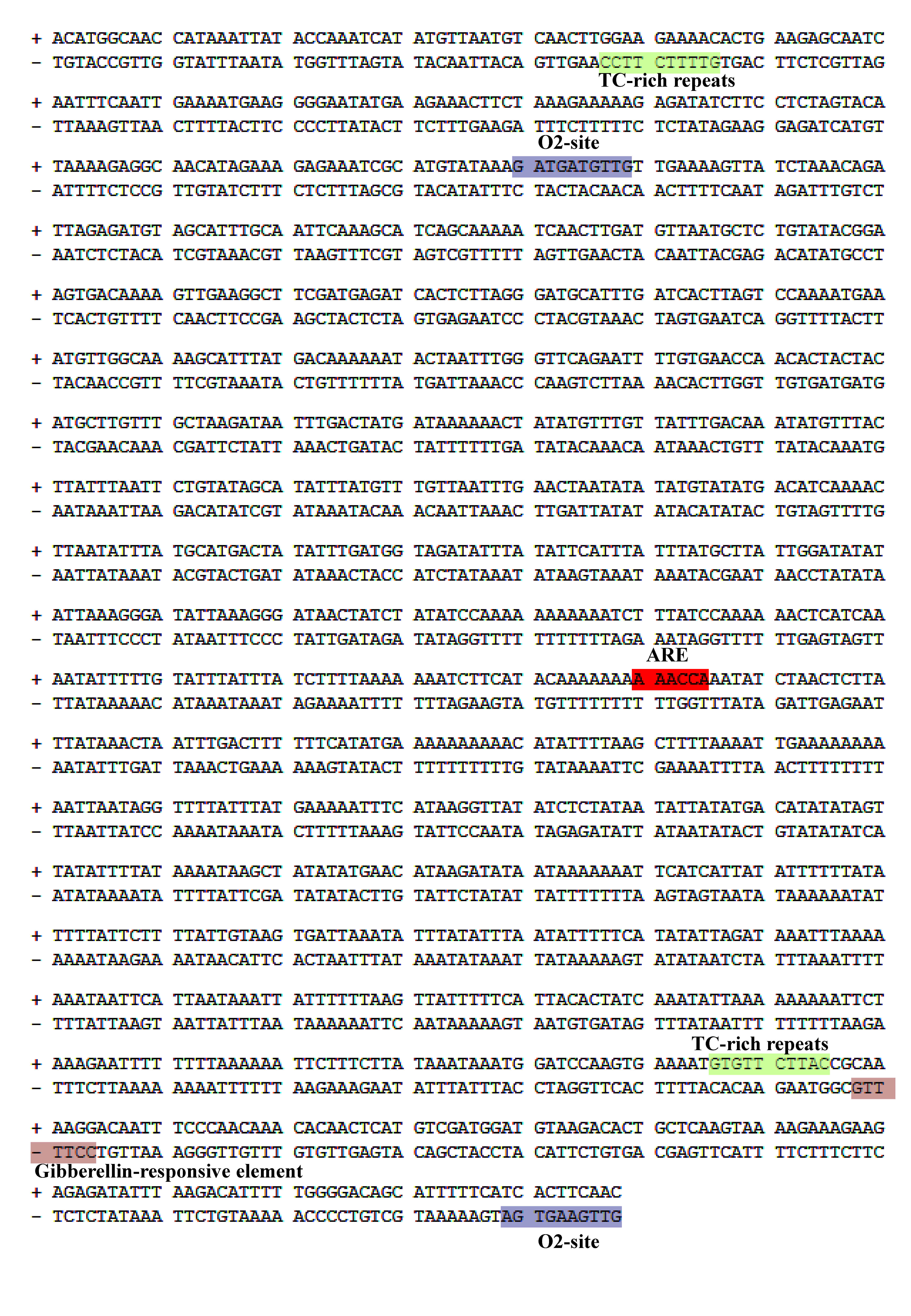

Supplement: Supplementary file 1 [file ijms-24-09354-s001.zip › Supplementary Figure S1.tif]
